# Effect of Fermentation on the Quality of Dried Hollow Noodles and the Related Starch Properties

**DOI:** 10.3390/foods11223685

**Published:** 2022-11-17

**Authors:** Xue Lu, Xiaona Guo, Kexue Zhu

**Affiliations:** 1 State Key Laboratory of Food Science and Technology, Jiangnan University, Wuxi 214122, China; 2 School of Food Science and Technology, Jiangnan University, Wuxi 214122, China

**Keywords:** *Lactobacillus plantarum* pre-fermentation, yeast fermentation, Koji pre-fermentation, starch characteristics, machine-made dried hollow noodles

## Abstract

Crumbly dough fermentation was applied to produce dried hollow noodles, with *Lactobacillus plantarum*, Koji and yeast as the main fermenting agents. The cooking, textural and digestive properties of the noodles were studied, followed by the morphological, crystalline and thermal properties of the starch. The results show that, compared to unfermented noodles, the optimal cooking time of Koji pre-fermented noodles (KJHN) decreased from 460 s to 253 s, and they possessed a higher percentage of weakly bound water and degree of gelatinization at the same cooking time. After cooking, KJHN had a softer texture and higher starch digestibility. In addition, the physicochemical properties of the KJHN and *Lactobacillus plantarum* pre-fermented noodles (LPHN) showed a decrease in pH and amylose content, and an increase in reducing sugars content. The starch extracted from KJHN and LPHN had significant superficial erosion and pore characteristics, and the gelatinization enthalpy, relative crystallinity and short-range order were all increased. These changes in the starch properties and the quality characteristics of noodles resulting from Koji fermentation might provide a reference for the development of easy-to-cook and easy-to-digest noodles.

## 1. Introduction

As one of the staple foods, noodles have a long history in China. Among them, the hand-made dried hollow noodles are naturally fermented and have a porous cross-section, originating from Shaanxi province in China. Hollow noodles are easy to cook and digest, have a soft and elastic texture and are especially popular for the elderly, pregnant women and infants. The production of hand-made hollow noodles consists of more than a dozen processes, including repeated kneading, dividing, relaxing, hanging, pulling and, finally, naturally drying. Since the whole process is done by hand, it is time-consuming and labor-intensive. In order to simplify the production process while retaining the quick cooking characteristics and soft texture characteristics, machine-made dried hollow noodles were introduced.

The common production method is to dissolve yeast in water and mix it with flour to form a crumbly dough, and then the final product is obtained through the operations of relaxing, sheeting, cutting, noodle fermenting and machine air-drying. All procedures can be done independently by mechanical equipment, with little or no human involvement. Xiong et al. [1] concluded that machine-made hollow noodles produced from flour containing a higher protein content had a relatively shorter cooking time and better textural quality, and appropriate yeast addition and suitable noodle fermentation time helped to increase the porosity of hollow noodles. On the basis of using yeast as the starter culture, Ge et al. [2] introduced *Lactobacillus plantarum* to ferment the noodles, which increased the pore structure of the noodles and shortened the cooking time. The porous structure formed by the fermentation not only gave the noodles a shorter cooking time, but also effectively reduced the hardness of the noodles after cooking. At the same time, the production process of machine-made hollow noodles greatly simplified the manufacturing process and saved the manpower workload. Fermentation is a complex and slow process; microbial metabolism may alter or degrade the starch after a long fermentation time, which affects its water absorption and swelling properties during cooking, which in turn affects the cooking time of the noodles and ultimately results in different textural properties. Li et al. [3] concluded that the co-fermentation of *Lactobacillus* and yeast resulted in the rice noodle having a crisper and softer texture, which was explained by the fact that the microbial fermentation disturbed the crystalline order of the rice starch, breaking up the starch and allowing it to swell more easily during processing. Zhu et al. [4] used mixed *Lactobacillus* to ferment rice flour; organic acids and enzymes were produced in the system, which might hydrolyze starch to some extent, thus shortening the rehydration time of rice noodles. In addition, a large amount of amylose leached, thus forming a strong network structure, which led to the increase in the hardness and chewiness of the rice noodles.

At present, the production process of machine-made dried hollow noodles has not been well established and is still in the stage of exploration and innovation. The existing literature has mainly investigated the use of yeast for noodle fermentation, while pre-fermentation of the crumbly dough and Koji fermentation have not been reported for the production of machine-made hollow noodles. Therefore, in this study, different fermentation methods were used to make noodles in order to find a suitable method for the production of hollow noodles with a shorter cooking time, softer texture and higher in vitro digestibility, thus satisfying the current pursuit of a fast pace of life and providing recommendations for the development of diets for specific groups of people such as the elderly, children and pregnant women.

The main objectives in this study were as follows: (1) to study the effect of the fermentation formulation on the cooking properties, textural and chemical properties of hollow noodles; (2) to study the water absorption properties and gelatinization properties during cooking, and in vitro digestive properties after cooking; (3) to study the morphological properties, thermal properties and crystallization properties of the starch isolated from dried noodles using scanning electron microscopy (SEM), differential scanning calorimetry (DSC), X-ray diffractometry (XRD) and Raman spectroscopy.

## 2. Materials and Methods

### 2.1. Materials

The wheat flour was provided from COFCO Corporation (Qinhuangdao, China) (containing 15.50% protein, 12.00% moisture, 0.44% ash); glucose oxidase kit (F006-1-1, Nanjing Jiancheng Bioengineering Institution); pepsin (≥250 U/mg, 9001-75-6), pancreatic enzymes (USP × 8; trypsin ≥ 200 U/mg, 8049-47-6), alpha-amylase (≥60 U/mg, 10,080) were purchased from Sigma; amyloglucosidase (≥100 U/mg, 9032-08-0) was purchased from JK Chemical; commercial yeast and Koji were purchased from Angel Yeast Co. Ltd. (Yichang, China); commercial *Lactobacillus plantarum* powder was purchased from Zhenjiang Tianyi Biotechnology Co., Ltd. (Zhenjiang, China). All chemicals were analytical grade.

### 2.2. Preparation for Noodles

Unfermented noodles (control): 400 g of flour and 128 g of water were mixed using a needle dough mixer (JHMZ, Beijing, China) and then equilibrated at 30 °C and 75% humidity for 30 min. After that, the crumbly dough was pressed to 0.8 mm from 2 mm, 1.6 mm and 1.2 mm by a noodle machine (JMTD-168/140, Beijing, China), then cut into 2.3 mm width, dried in a noodle drying machine to 13 ± 0.5% moisture (SYT-030, Beijing, China) and, finally, packed into plastic bags for later analysis. Unfermented noodles were used as the control for this experiment.

Hollow noodles 1 (HN1): 4 g yeast was dissolved in 128 g water and then mixed with 400 g flour. After relaxing, sheeting and cutting, the noodles were fermented at 30 °C and 75% humidity for 90 min, then dried and packaged. Yeast was used in this recipe for noodle fermentation.

Hollow noodles 2 (HN2): 2 g yeast was dissolved in 64 g water, then mixed with 200 g flour to form a crumbly dough, fermented at 30 °C and 75% humidity for 24 h, and then mixed with 200 g flour containing 2 g yeast and 64 g water. After relaxing, sheeting and cutting, the noodles were fermented at 30 °C and 75% humidity for 90 min, then dried and packaged. In this recipe, yeast was used for crumbly dough pre-fermentation and noodle fermentation.

*Lactobacillus plantarum*-fermented hollow noodles (LPHN): 9 g *Lactobacillus plantarum* powder was dissolved in 64 g water, then mixed with 200 g flour to form a crumbly dough, fermented at 30 °C and 75% humidity for 24 h, and then mixed with 200 g flour containing 2 g yeast and 64 g water. After relaxing, sheeting and cutting, the noodles were fermented at 30 °C and 75% humidity for 90 min, then dried and packaged. In this recipe, *Lactobacillus plantarum* was used for crumbly dough pre-fermentation.

Koji fermented hollow noodles (KJHN): 3 g koji powder was dissolved in 64 g water, then mixed with 200 g flour to form a crumbly dough, fermented at 30 °C and 75% humidity for 24 h, and then mixed with 200 g flour containing 2 g yeast and 64 g water. After relaxing, sheeting and cutting, the noodles were fermented at 30 °C and 75% humidity for 90 min, then dried and packaged. In this recipe, Koji was used for crumbly dough pre-fermentation.

### 2.3. Analysis of Cooking Properties

The cooking characteristics of the noodles mainly include optimal cooking time, water absorption and cooking loss, which were determined according to the method by Chillo et al. [5] with appropriate modifications.

Optimal cooking time: 25 g of noodles were placed in 500 mL of boiling water. One noodle was taken out every 15 s and placed in two transparent glass plates and squeezed. The time when the white core in the middle of the noodle just disappeared was the optimal cooking time.

The water absorption of dried noodles cooked for 3, 5 and 7 min was calculated by the following formula:

Water absorption (%) = ((M1 − M2)/M2) × 100

where M1 represents the weight of the noodles after cooking, and M2 represents the weight of the dried noodles before cooking.

The cooking water at the optimal cooking time was collected and the volume fixed to 500 mL with deionized water. Then, 100 mL was taken out in a constant weight beaker, heated on an infrared oven and then placed in a 105 °C oven for constant weight, and the cooking loss was calculated using the following formula:

Cooking loss (%) = (m × 5/M) × 100

where M is the weight of dried noodles before cooking (g), and m is the weight of insoluble matter in the beaker (g).

### 2.4. Analysis of Texture Properties

The texture properties were determined by the method of Han et al. [6] with some modifications.

TPA (texture profile analysis) mainly included hardness, springiness and chewiness of the noodles after cooking by a TA-XT2i Texture Analyzer (Stable Micro Systems, London, England). The noodles were cooked for the optimum cooking time, then three noodles were placed on the platform using a P36/R probe to simulate the chewing test with the following test parameters: pre-test velocity, 2.0 mm/s; test velocity, 1.0 mm/s; post-test velocity, 2.0 mm/s; strain, 75%; trigger force, 5.0 g.

Tensile properties analysis was determined by measuring the tensile force and distance of the cooked noodles. Noodles were cooked for the optimal cooking time and tensile measurements were carried out using the A/SPR probe until the noodles broke with the following test parameters: pre-test velocity, 1.0 mm/s; test velocity, 2.0 mm/s; post-test velocity, 2.0 mm/s; distance, 150 mm. trigger force, 5.0 g.

Both TPA and tensile analysis were measured 10 times in parallel.

### 2.5. Chemical Properties of Noodles

#### 2.5.1. pH

The pH of the noodles was determined by using a pH meter (STARTER 3100/F, Shanghai, China). This was done by taking 10 g of noodles and adding 90 mL of deionized water, magnetic stirring for 30 min and then measuring.

#### 2.5.2. Reducing Sugar

Reducing sugar was determined according to the method of Su et al. [7]. Approximately 4 g (based on dry weight) of noodle flour were added to a centrifuge tube containing 30 mL of 0.2% calcium chloride solution, followed by magnetic stirring for 60 min and then centrifuged at 4000 rpm/min for 10min. The supernatant was fixed to 50 mL with deionized water. 1 mL was diluted by adding 13 mL of deionized water, then 1 mL was taken and added to 2 mL of DNS in a water bath at 99 °C for 10 min. The absorbance was measured at 540 nm using a spectrophotometer (TU-1810, Beijing, 210 China). Blank samples were obtained by replacing 1ml of sample with 1ml of deionized water. The reducing sugar content was based on the maltose content and was calculated using the following formula:

Reducing sugar content (mg/g) = m × d/M

where M is the sample mass (dry weight/g), m is the amount of reducing sugars (mg), and d is the dilution factor.

#### 2.5.3. Amylose Content

The amylose content of the starch was determined by the Megazyme amylose/amylopectin assay procedure (Megazyme International, Bray, Ireland) using a selective precipitation reaction of concomitant cuttlefish globulin A (Con A) with amylopectin starch [8].

### 2.6. Analysis of Water Distribution

The water distribution characteristics were determined by the method of Yang et al. [9] with appropriate modifications. The surface water of noodles cooked for 5 min was drained with filter paper, and then 1 g of noodles was put into an NMR tube with a sealing film at the mouth of the bottle to prevent water loss. For the NMR experiments, the Carr–Purcell–Meiboom–Gill (CPMG) pulse sequence was used with the following parameters: scan frequency (SF) = 21 kHz, number of sampling points (TD) = 112490, sampling interval (TW) = 2000 ms, number of repetitive scans (NS) = 2, echo time (TE) = 0.25 ms, the number of echoes (NECH) = 4500.

### 2.7. Degree of Gelatinization of Noodles

The determination of the degree of gelatinization was based on the method of Liu et al. [10] with appropriate modifications. The freeze-dried noodle powder for 3, 5 and 7 min cooking was fully dissolved with different concentrations of alkali and then hydrolyzed using amyloglucosidase, after which the absorbance of the sample was measured by spectrophotometer (TU-1810, Beijing, China) at 510 nm using a glucose oxidase kit and then converted to a standard glucose concentration.

### 2.8. Starch Digestibility (In Vitro) of Noodles

In vitro digestion of cooked noodles was measured according to Minekus et al. [11], with appropriate modifications. The whole digestion process was divided into three parts: oral, gastric and intestinal. The process was as follows: firstly, the noodles were cooked to the optimal cooking time, and then 5 g of noodles were taken in a centrifuge tube with the following steps in sequence.

Oral digestion (2 min): 1 mL of α-amylase (pH = 4.7), 25 μL of calcium chloride (0.3 M), 4 mL of sodium acetate buffer solution (ABS) (0.1 M, pH = 7) were added to a centrifuge in order to make the final α-amylase enzyme activity of 75 U/mL.

Gastric digestion (pH = 2, 2 h): 1.3 mL HCl (1 M), 1.6 mL pepsin (pH = 2), 5 μL calcium chloride (0.3 M), 7.1 mL ABS (0.1 M, pH = 7) were added sequentially, making the final pepsin enzyme activity of 2000 U/mL.

Intestinal digestion (pH = 7, 3 h): 1.4 mL NaOH (1 M), 5 mL pancreatic enzyme, 40 μL calcium chloride (0.3 M), 13.6 mL ABS (0.1 M, pH = 7) were added sequentially to make the final pancreatic protease enzyme activity of 100 U/mL.

Inactivation and determination: 0.1 mL of the reaction solution removed at 0, 20, 60, 120, 180 and 240 min was mixed with 0.3 mL of 100% ethanol, vortexed for 30 s to inactivate the enzyme and centrifuged at 10,000× *g* for 10 min. 0.1 mL of the supernatant (0.1 mL of deionized water for control) was mixed with 0.5 mL amyloglucosidase (pH = 4.7, 0.1 M ABS), and the reaction was shaken for 60 min at 37 °C. Then, the glucose content was determined using the glucose oxidase kit.

Starch digestibility (%) = ((GB−GA) × C × 0.9/M) × 100

where GB is the glucose content of experimental group, GA is the glucose content of the control group, C is the dilution factor, 0.9 is the conversion coefficient of glucose to starch, and M is the total starch content. The obtained data were then fitted with first-order rate equation [12].

y = a × (1 − exp(−b × t))

where y is the starch hydrolysis rate, a is the predicted endpoint starch digestibility, and b is the starch digestibility coefficient. The value of R2 was used to verify the credibility of the digestion data, after which a line graph of starch digestibility in vitro with time was plotted.

### 2.9. Isolation of Starch from Dried Noodles

The starch was extracted according to the method of Noda et al. [13], with appropriate modifications. The method was as follows:

The sample was soaked for 60 min in a beaker with deionized water and then washed by hand, centrifuged at 5000 rpm for 10 min and the supernatant and yellow precipitate were discarded. This operation was repeated three times until no yellow precipitate was visible. The white precipitate was then dispersed in 40 mL of water and sieved through a 75 μm sieve. The filtrate was collected, centrifuged, and the lower precipitate was starch. The precipitate was washed with 70% ethanol, centrifuged and repeated twice. Finally, the sample was dehydrated in 100% ethanol and dried in an oven at 40 °C for 24 h.

### 2.10. Scanning Electron Microscopy (SEM)

The microstructures of starch were observed using a scanning electron microscope (SU8100, Hitachi Int., Tokyo, Japan) with an accelerating voltage of 3.0 kV. The samples were sprinkled in appropriate amounts on conductive adhesive and then sprayed gold.

### 2.11. Differential Scanning Calorimetry (DSC)

The thermal properties of starch were determined by reference to the method of Ding et al. [14]. DSC 3 (Mettler Toledo, Schwerzenbach, Switzerland) was used to determine the thermal properties of starch. Starch samples (5 ± 0.03 mg, dry starch basis) and 15 μL of deionized water were added to 40 μL of standard aluminum pans; then it was sealed and equilibrated overnight at room temperature. The scanning temperature was 30–100 °C and the heating rate was 10 °C/min. The enthalpy change (ΔH), starting (To), peak (Tp) and end (Tc) temperatures were calculated using STARe evaluation software (Version 15.0, Mettler Toledo, Schwerzenbach, Switzerland).

### 2.12. X-ray Diffractometry (XRD)

The XRD determination of starch was carried out according to the method of Zhao et al. [15] by X-ray diffractometer (D2 PHASER, Bruker AXS GMBH, Germany). The relative crystallinity of starch was determined by using the following measurement conditions: target type Cu Kα, voltage of 30 kV, scan speed of 2 °/min, scan range 2θ of 4 to 35° and a scan step of 0.02°. The relative crystallinity was calculated using JADE 6.0 (Materials Date Inc., Livermore, CA, USA).

### 2.13. Laser Confocal Micro-Raman (LCM-Raman) Spectroscopy

Raman spectroscopy was performed using LCM-Raman (LabRAM HR Evolution, HORIBA Jobin Yvon S.A.S., Palaiseau, France) with a 532 nm green diode laser source. The starch samples were laid flat on tinfoil-covered slides and the spectra were collected in the range of 3200–100 cm^−1^ with a magnification of 50 × LWD, an integration time of 4 s and a cumulative count of 3 times. The full width at half maximum (FWHM) of the band at 480 cm^−1^, which was used to analyze the short-range molecular order of starch, was calculated using Origin 2021 software (OriginLab, Northampton, MA, USA).

### 2.14. Statistical Analysis

All experiments were performed at least three times, and results were expressed as mean ± standard deviation. Significance analysis was performed using the one-way analysis of variance (ANOVA) of SPSS (SPSS Inc., Chicago, IL, USA) software, and plotting was performed using Origin 2021 (OriginLab).

## 3. Discussion

### 3.1. Effect of Fermentation Methods on Cooking Properties of Dried Hollows Noodles

Cooking time and cooking loss are important quality indicators of dried noodles, which affect the degree of acceptance by consumers. The cooking properties of dried hollow noodles prepared by different fermentation methods are shown in Table 1. Compared with the optimal cooking time of unfermented noodles (Control, 460 s), hollow noodles 1 (HN1), which only underwent noodle fermentation, reduced the time to 407 s. The possible reason was that the gas produced by microbial metabolism during the noodle fermentation formed many pores inside the noodles (Table 1), which were conducive to the contact between water and starch during cooking, thus shortening the cooking time of HN1. The optimal cooking time of hollow noodles 2 (HN2) was 332 s, which underwent fermentation of both the crumbly dough and noodles. The possible reason is not only the porous structure produced in the process of noodle fermentation, but also the hydrolysis of starch by microbial metabolites during the long (24 h) fermentation time of the crumbly dough, which might be beneficial to the water absorption, expansion and gelatinization of the starch during noodle cooking, thus making HN2 have a shorter cooking time. In addition, *Lactobacillus plantarum*-fermented hollow noodles (LPHN) and Koji-fermented hollow noodles (KJHN), which also underwent the pre-fermentation of the crumbly dough and noodle fermentation, reduced the optimal cooking time to 267 s and 253 s. The possible reason is that the addition of *Lactobacillus plantarum* and Koji had a greater effect on the properties of the starch during the long pre-fermentation time of the crumbly dough compared with HN2, thus conferring a shorter cooking time to the hollow noodles. The KJHN had the shortest cooking time, which might be more in line with people’s demand for noodles that can be cooked in a short time.

From the results in Table 1, it can be concluded that, compared with unfermented noodles (control), hollow noodles1 (HN1,) which only experienced noodle fermentation and hollow noodles 2 (HN2), which experienced both crumbly dough pre-fermentation and noodle fermentation, had relatively low cooking loss, which might be explained by the relatively short cooking time. In contrast, the LPHN and KJHN obtained by replacing yeast with *Lactobacillus plantarum* and Koji during the crumbly dough fermentation possessed relatively high cooking losses at a shorter cooking time, probably due to some hydrolysis of the starch by microorganisms or their metabolites during the long fermentation time of the crumbly dough, which affected the structural properties of the starch and resulted in the leaching of amylose during cooking [16]. In addition, the long *Lactobacillus plantarum* pre-fermentation might also cause hydrolysis of gluten [17], thus making the starch molecules more likely to spill out during cooking and thus exhibit higher cooking losses; this property might also facilitate the contact between the starch and enzymes, thus having some effect on the in vitro digestion of starch.

### 3.2. Effect of Fermentation Methods on the Texture Properties of Cooked Noodles

The texture properties included the TPA (texture profile analysis) and tensile test of the cooked noodles, which are commonly used to characterize the eating quality of noodles. From the results in Table 1, it can be seen that fermentation generally reduced the hardness and chewiness of the noodles, and the springiness remained essentially unchanged. Compared to unfermented noodles (control), HN1 showed lower firmness and chewiness, probably because the carbon dioxide produced by the yeast during noodle fermentation formed porous structures in the noodles, causing them to absorb more water during cooking, thus showing lower firmness. The HN2 group, which had both crumbly dough and noodle fermentation, had a slightly higher hardness than HN1; the possible reason might be that the metabolites produced by the 24 h yeast pre-fermentation inhibited the gas production of the yeast during the fermentation of the noodles, which thus exhibited a lower porosity. The appearance properties in Table 1 just confirmed this hypothesis. *Lactobacillus plantarum*-fermented hollow noodles (LPHN) and Koji-fermented hollow noodles (KJHN) also exhibited lower firmness and chewiness, the reason for which might be attributed to the metabolic gas production by yeast using sugars and the hydrolysis of starch or protein by metabolites, which in turn affected the textural properties of the noodles by the affecting water absorption capacity of the noodle matrix during cooking. Similar results were obtained by Baik et al. [18], where noodles with a low amylose content showed a softer texture when cooked, even with an increased protein content.

As could be seen from Table 1, the tensile properties of the noodles after fermentation mainly showed a decrease in tensile breaking force, and the overall change in tensile distance was not particularly obvious, with the KJHN group pre-fermented with Koji having the lowest breaking force and showing softer texture characteristics. The yeast fermentation groups (HN1 and HN2) exhibited lower forces, probably due to the discontinuous structural characteristics that appeared inside the porous noodle matrix after cooking, which showed easier fracture in the tensile experiments. The *Lactobacillus plantarum* and Koji pre-fermentation groups (LPHN and KJHN) exhibited the lower breaking force, probably due to a series of microbial metabolic behaviors during fermentation that not only produced a porous structure but also might have affected the starch or protein properties, thus affecting the tensile properties of the noodles after cooking. Xu et al. [16] concluded that the fermented noodles exhibited a significant decrease in tensile properties, which could be attributed to the internal structural changes in dried noodles. In addition, the soft and elastic characteristics of KJHN and LPHN might be favored by people with dental problems, such as babies and the elderly.

### 3.3. Effect of Fermentation Methods on the Chemical Properties of Noodles

#### 3.3.1. pH

According to the results of Table 1, compared with the pH of the unfermented control (6.04), the pH of fermented dried noodles decreased to different degrees, among which, HN1, which only underwent noodle fermentation, reduced the pH to 5.83; HN2, which underwent both crumbly dough and noodle fermentation, reduced the pH to 5.20; LPHN, which replaced yeast with *Lactobacillus plantarum*, reduced the pH to 4.02; and KJHN, which replaced yeast with Koji, reduced the pH to 5.67. The reason for the decrease in pH values of un-pre-fermented HN1 and yeast pre-fermented HN2 might be that acidic substances such as succinic acid were produced during fermentation [19], and the acidic substances accumulated with the extension of time. In addition, the decrease in pH of the Koji pre-fermented KJHN might be attributed to the decomposition of sugars in the fermentation process to produce acidic substances such as malic acid and succinic acid [20]. The decrease in pH of *Lactobacillus plantarum* pre-fermented LPHN might be due to *Lactobacillus plantarum*-produced acids such as lactic acid and acetic acid during fermentation [21]. Moreover, these acidic substances might affect starch to some extent, which then affects the water distribution and gelatinization behavior of dried noodles during cooking.

#### 3.3.2. Reducing Sugars

In the fermentation process, microorganisms use reducing sugar for respiration and, on the other hand, they decompose substrates to produce reducing sugar. Therefore, by detecting the content of reducing sugar, the degree of fermentation can be predicted. From the Table 1 results, it can be obtained that, compared with the unfermented noodles (control), the HN1 (without pre-fermentation) and HN2 (yeast pre-fermentation) showed a significant decrease in reducing sugars (*p* < 0.5), while the LPHN (*Lactobacillus plantarum* pre-fermentation) and KJHN (Koji pre-fermentation) showed a significant increase in reducing sugars (*p* < 0.5). Where HN2 had a lower reducing sugar content than HN1, the possible reason is that the rate of reducing sugar production was lower than the rate of reducing sugar utilization during the long fermentation time. In contrast, LPHN had a higher amount of reducing sugars, probably due to some acidolysis of starch by microorganisms during the fermentation process, as it also had a lower pH [22]. Moreover, the main microorganism in the KJHN fermentation group is Rhizopus, which could produce amylase and amyloglucosidase [23], and, therefore, some hydrolysis of the starch might have occurred in this fermentation experiment; similar results were reported by Wu et al. [24], where Rhizopus used carbon sources to produce organic acids, sugars, ethanol and carbon dioxide during the fermentation of oats.

#### 3.3.3. Amylose Content

From the data in Table 1, it can be seen that there was no significant difference in the amylose content among the control, HN1 and HN2, but LPHN and KJHN had a significantly lower content (*p* < 0.05). HN1 and HN2 were both yeast-fermented, in which HN2 underwent a long crumbly dough pre-fermentation. Yeast mainly metabolizes sucrose and raffinose by intracellular transposase [25]; although some acids might accumulate in the metabolic process, it might not be enough to affect the fine structure of starch. The *Lactobacillus plantarum* pre-fermentation group (LPHN) and Koji pre-fermentation group (KJHN) exhibited a lower amylose content, probably due to the production of acids/enzymes capable of hydrolyzing starch during the fermentation process, which always starts from the amorphous region rich in amylose. Similar results were reported by Reyes et al. [26], where microorganisms hydrolyzed the amorphous area more readily than the crystalline area. The results of Xu et al. [27] showed that lactic acid bacteria hydrolyzed the amorphous area of starch, causing the breakdown of some amylose starch into dextrin and monosaccharides, resulting in a significant decrease in amylose content. In addition, the amylose content had a great positive correlation with hardness and cooking time, which might be related to the looseness of the amorphous region, affecting the gelatinization process of starch during cooking.

### 3.4. Effect of Fermentation Methods on the Water Absorption Characteristics and Gelatinization Degree of Noodles during Cooking

#### 3.4.1. Water Absorption Characteristics

**Figure 1:**
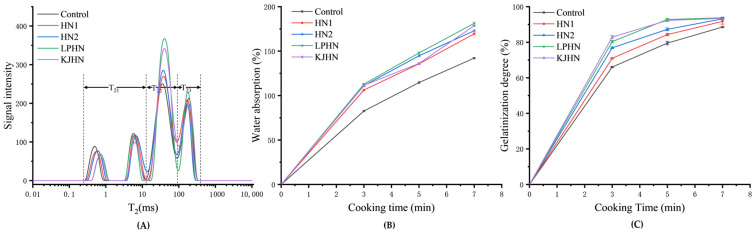
Effect of fermentation methods on the water distribution characteristics (**A**,**B**) and degree of gelatinization of noodles (**C**) during cooking. Control, unfermented noodles; HN1, hollow noodles 1; HN2, hollow noodles 2; LPHN, *Lactobacillus plantarum* fermented hollow noodles; KJHN, Koji fermented hollow noodles.

The cooking process of dried noodles is a process of starch gelatinization and cross-linking polymerization of gluten, and water plays an important role in this process. From Figure 1B, it can be obtained that the water absorption rate of the noodles gradually increased with the extension of the cooking time, and that of the fermented noodles (HN1, HN2, LPHN and KJHN) was generally higher than that of the unfermented noodles (control); the possible reason might be that the porous structure in the fermented noodles tended to retain more water during the cooking process. Among them, the *Lactobacillus plantarum* group (LPHN) consistently possessed a higher water uptake rate during cooking, and based on its lower pH (4), it might be speculated that the acidic hydrolysis made it easier for starch or protein to hydrate with water molecules. When cooked for 5 min, the water uptake rate and optimal cooking time varied between noodles. Compared to the unfermented control, the LPHN group possessed the highest water uptake rate, followed by HN2, KJHN and HN1. In order to understand the distribution characteristics of water during the cooking process, the noodles cooked 5 min were tested by LF-NMR experiments and the results are shown in Figure 1A. From the results in Figure 1A, it can be concluded that, at the same cooking time, the noodle group with a relatively shorter cooking time had lower A_21_ (strongly bound water, water inside the starch granules or water tightly bound to gluten proteins) and A_23_ (free water, freely movable water in the capillaries) values and higher A_22_ (weakly bound water, water outside the starch granules or inside the gluten network) values [28]; therefore, it could be speculated that A_22_ played an important role in the cooking process of dried noodles and might contribute to the water absorption, starch gelatinization and stretching behavior of the gluten. Zhang et al. [29] also reported that, in the process of treating rice flour with 1,4-α-glucan branching enzymes, with the increase in enzyme activity, the optimal cooking time of rice noodles was shortened. Therefore, at the same cooking time, the faster-cooked rice noodle group tended to absorb more water and had a higher proportion of weakly bound water, while the higher proportion of weakly bound water dispersed in the starch gel network structure was more conducive to the recovery of the elastic texture of the rice noodles.

#### 3.4.2. Gelatinization Degree of Noodles

In general, the degree of gelatinization of noodles could be used to determine the gelatinization state of the starch and the cooking state of the noodles. From Figure 1C, the degree of gelatinization of the noodles gradually increased with the extension of the cooking time, and the fermented noodles were always higher than the unfermented noodles (control), where the KJHN group had the highest value, followed by LPHN, HN2 and HN1. Moreover, from the results, we find that the noodles with a shorter cooking time always show higher gelatinization at the same cooking time. For fermented noodles, the porous structure produced by fermentation made it easier for water molecules to diffuse in the noodle matrix during cooking, and the starch present in the matrix was more likely to absorb water. Therefore, the fermented noodles possessed a higher degree of gelatinization compared to the control. Furthermore, it could be speculated from the results of Section 3.3 that, in the *Lactobacillus plantarum* and Koji pre-fermentation groups (LPHN and KJHN), acids and enzymes might have subjected the starch to hydrolysis, thus making it easier for water molecules to enter the amorphous growth ring structure of the starch and disrupt the starch crystallization zone, which in turn exhibited easier gelatinization [30].

### 3.5. Effect of Fermentation Methods on the Starch Digestibility (In Vitro) of Noodles

**Figure 2:**
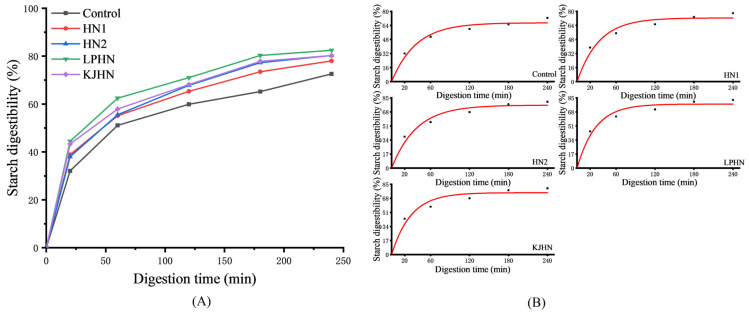
Effect of fermentation methods on the starch digestibility (in vitro) of noodles (**A**) and equation fitting diagram (**B**). Control, unfermented noodles; HN1, hollow noodles 1; HN2, hollow noodles 2; LPHN, *Lactobacillus plantarum*-fermented hollow noodles; KJHN, Koji-fermented hollow noodles.

The starch digestibility of the noodles is shown in Figure 2A. The R2 after fitting the equations (Figure 2B) were all greater than 0.95 and the data had good confidence. The starch digestibility of the fermented noodles was generally higher than that of the unfermented noodles (control). The LPHN group had the relatively highest in vitro starch digestibility, followed by HN2, KJHN and HN1. The discontinuity in the noodle substrate produced by fermentation gave the enzyme the opportunity to contact starch molecules, which led to a higher starch hydrolysis rate in vitro, which might explain the higher digestibility of fermented dried noodles. In addition, for the LPHN and KJHN groups, the amorphous region of the starch might be hydrolyzed, which in turn might lead to a lower content of amylose and long-branched starch and an increase in the relative content of short-branched starch, thus increasing the action site of the enzyme and the relative efficiency of the hydrolysis [27]. Moreover, the structural distribution of the noodles was that the starch was embedded in a protein matrix and a lower pH might cause proteolysis of the protein matrix surrounding the starch granules [21]. As a result, starch was released from the protein matrix and readily hydrolyzed, thus increasing the digestibility of the starch in the LPHN group. However, there was no significant difference in the in vitro digestibility of starch between the fermented noodles (overlapping polylines), probably because it was not only the starch that determined in vitro digestibility, but possibly other factors such as the distribution of pores produced by fermentation, the integrity of the protein network structure and the fragmentation degree of the noodle when simulating human chewing, etc. Moreover, fermented noodles are easy to digest, so they are more suitable for people with a high energy demand or imperfect digestive system, such as pregnant women or infants.

### 3.6. Starch Morphology by Scanning Electron Microscopy

**Figure 3:**
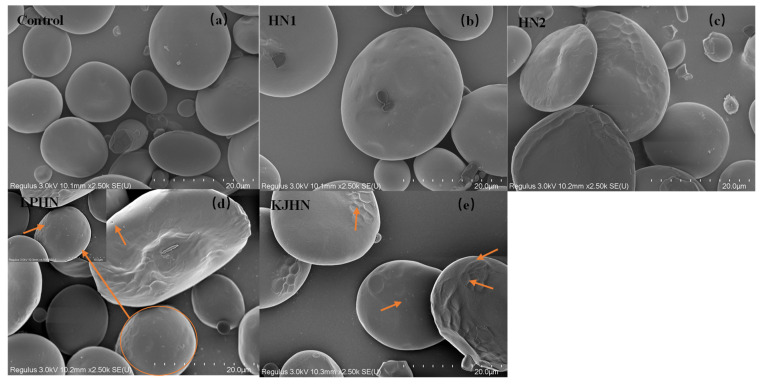
Scanning electron micrographs of starch. (**a**) Control, unfermented noodles; (**b**) HN1, hollow noodles 1; (**c**) HN2, hollow noodles 2; (**d**) LPHN, *Lactobacillus plantarum*-fermented hollow noodles; KJHN, (**e**) Koji-fermented hollow noodles.

The morphological properties of the starch isolated from noodles are shown in Figure 3. The surface of the starch from unfermented noodles (Control, a) and the starch from the HN group were smooth and slightly uneven, respectively, while the starch from the groups that had a long crumbly dough fermentation time (c, d, e) showed a more intense erosion pattern, with the LPHN group (d) and KJHN group (e) showing obvious pore structure, cracks and surface erosion characteristics. From the results of Section 3.3, it can be concluded that LPHN and KJHN exhibited a lower pH and higher reducing sugar production during fermentation, respectively. Therefore, it was presumed that acid/enzymatic hydrolysis might have occurred. Similar results were reported by Zhai et al. [31], where pitting properties were evident on the starch surface after the treatment of raw starch using amylose. Majzoobi et al. [32] used acetic and lactic acids to treat raw wheat starch, and the results from SEM images showed that acid corrosion formed cracks and spots on the starch surface. In addition, based on previous studies, there were two main ways of acid/enzymatic hydrolysis [33]: one was the formation of a lamellar scale structure on the surface of the starch granules, the other was centripetal hydrolysis. A pore structure was formed on the surface of the granule, which led to hydrolysis from the surface to the internal center of the granule. Thus, based on the different acid and enzyme production capacities of the fermentation, different degrees of hydrolysis were produced on the starch surface; therefore, the pictures from SEM showed different characteristics. Furthermore, these different degrees of hydrolysis might impart different gelatinization behaviors to the starch during cooking, which in turn affected the optimal cooking time or textural properties of the noodles.

### 3.7. Effect of Fermentation Method on the Starch Properties from Dried Noodles

#### 3.7.1. Thermal Properties

The results of thermal parameters of starch gelatinization are shown in Table 2, mainly including the starting temperature (To), peak temperature (Tp), end temperature (Tc) and enthalpy (ΔH). ΔH is the energy required to break the double helix in the crystalline and amorphous regions of starch during gelatinization. From the results in Table 2, it can be seen that the starch from Control, HN1 and HN2 showed no significant change in gelatinization temperature and enthalpy, while the LPHN and KJHN groups slightly increased To, Tp and Tc, and significantly increased the enthalpy (*p* < 0.05). Combining the results of SEM and the reduced amylose content, it can be inferred that, in the fermentation process of KJHN and LPHN, acids or enzymes hydrolyzed starch, especially in the amorphous area, which manifested itself as a more crystalline area when weighing the same weight of sample during DSC testing, thus requiring more thermal energy to disrupt the double-helix structure and crystalline area. A similar result was obtained by Al-Ansi et al. [22], where the enthalpy of naturally fermented barley starch reached a maximum at 24 h under the hydrolysis of enzymes and acids, and then started to decrease because of the transition of hydrolysis from the amorphous area to the crystalline area.

#### 3.7.2. Crystalline Properties

From the results of Table 2, it was observed that there was no significant change in the relative crystallinity of starch from Control, HN1 and HN2, while there was a significant increase in the relative crystallinity of starch from LPHN and KJHN (*p* < 0.05). The possible reason is that, during fermentation, *Lactobacillus plantarum* (LPHN) and Rhizopus (KJHN) produced acids and enzymes, which hydrolyzed the amorphous region rich in amylose [22], thus affecting the long-range order of the starch; this trend is consistent with the increasing enthalpy of DSC. Similar results were reported in previous studies [32,34], where acids and enzymes hydrolyzed the amorphous area and increased the relative crystallinity. In a study by Xu et al. [27], during the fermentation of starch with lactobacilli, the relative crystallinity of the starch increased during the first 42 h, while by 72 h, the relative crystallinity began to decrease as acids or amylase acted on the loosely branched starch outside the crystalline area, causing damage to the crystalline region. Therefore, it could be inferred that 24 h crumbly dough fermentation might not be enough to change the internal crystal structure of starch, and enzyme/acidolysis mainly affects the amorphous region of starch because the amorphous region of starch is rich in amylose, and enzyme/acidolysis tends to hydrolyze amylose [15]. HN2, which also underwent 24 h of crumbly dough fermentation, did not show a more pronounced increase in relative crystallinity, probably because the enzymes produced by the yeast were mainly intracellular [25], and the erosive effect of the produced acids [35] on starch might not be sufficient to be detected by the instrument. Therefore, the starch from HN1, which only underwent 90 min of noodle fermentation, was less likely to produce changes that could be detected by the instrument.

#### 3.7.3. Short-Range Ordered Structure

The characteristic spectral band at 480 cm^−1^ originates from the sensitivity of the glucopyranose ring structure to changes in the short-range molecular order of the double helix in starch. In particular, the area and intensity of the full width at half maximum (FWHM) and 480 cm^−1^ spectral bands were negatively correlated with the crystallinity of the starch. Many studies have shown that the FWHM of the 480 cm^−1^ spectral band is a good indicator of the short-range order degree in crystalline starches, and the narrower the FWHM, the higher the degree of the short-range order [36]. From the results in Table 2, it was observed that the FWHM values of starch from unfermented noodles and those fermented only with yeast (HN1 and HN2) did not change significantly, while the FWHM values of starches from the LPHN and KJHN groups that underwent 24 h crumbly dough fermentation decreased slightly, which might mean that the short-range order of the amorphous region of starch increased; this result is consistent with the trend of decreasing amylose content. Similar results were obtained in a study by Al-Ansi et al. [22], where microbial fermentation led to an increase in the proportion of short chains in the starch structure and in the interaction forces between short-chain molecules, resulting in the formation of more ordered crystalline starches between branched chain molecules, which led to an increase in short-range ordering.

## 4. Conclusions

This experiment applied the crumbly dough fermentation to produce dried hollow noodles with *Lactobacillus plantarum*, Koji and yeast as the main fermenting agents. Among these fermentation methods, hollow noodles fermented with Koji had a shorter cooking time, lower pH, lower amylose content and higher amount of reducing sugars. The Koji-fermented noodles after cooking were softer and had higher in vitro starch digestibility. In further studies of starch-related properties, the surface of the starch possessed corrosive and porous properties (from the SEM results) and the enthalpy of gelatinization, relative crystallinity and short-range orderliness of starch increased. This series of changes also allowed water to enter the interior of the noodles and starch more easily during cooking, resulting in noodles with higher percentages of weakly bound water and degree of gelatinization, as well as a shorter cooking time. The advantages of a short cooking time, soft and elastic texture, and easy digestibility after cooking of Koji-fermented hollow noodles might provide a reference for the development of diets suitable for infants, the elderly and pregnant women.

## Figures and Tables

**Table 1:** Cooking, texture and chemical properties and appearance of dried noodles.

Parameters	Samples				
	Control	HN1	HN2	LPHN	KJHN
Cooking properties					
CT/s	460.00 ± 0.00 ^a^	407.50 ± 3.54 ^b^	332.50 ± 3.54 ^c^	267.50 ± 3.54 ^d^	252.50 ± 3.54 ^d^
CL/%	5.22 ± 0.11 ^a^	4.25 ± 0.02 ^b^	4.01 ± 0.05 ^c^	6.81 ± 0.11 ^d^	5.17 ± 0.12 ^a^
Texture properties					
Hardness/N	50.49 ± 1.93 ^c^	40.68 ± 3.07 ^ba^	46.48 ± 2.46 ^ce^	40.47 ± 0.65 ^a^	38.99 ± 2.28 ^da^
Springiness/%	16.80 ± 0.08 ^a^	19.35 ± 0.13 ^b^	17.35 ± 0.25 ^a^	15.52 ± 0.58 ^c^	16.76 ± 0.62 ^a^
Chewiness/N	1.58 ± 0.02 ^c^	1.49 ± 0.05 ^a^	1.45 ± 0.03 ^a^	1.19 ± 0.12 ^b^	1.16 ± 0.04 ^b^
Tensile Properties					
Force/N	28.14 ± 1.50 ^a^	22.54 ± 2.00 ^b^	24.53 ± 0.42 ^c^	16.14 ± 0.51 ^d^	12.75 ± 1.17 ^e^
Distance/mm	55.62 ± 1.75 ^a^	43.93 ± 7.5 ^b^	56.33 ± 3.94 ^a^	54.27 ± 7.58 ^a^	44.50 ± 9.39 ^b^
Chemical Properties					
pH	6.04 ± 0.06 ^e^	5.83 ± 0.04 ^a^	5.20 ± 0.01 ^b^	4.02 ± 0.03 ^c^	5.67 ± 0.03 ^d^
Reducing sugar/(mg/g)	72.40 ± 0.46 ^e^	54.45 ± 0.16 ^a^	35.49 ± 0.47 ^b^	184.78 ± 0.00 ^c^	203.83 ± 1.66 ^d^
Amylose content/%	26.30 ± 0.15 ^b^	25.96 ± 0.10 ^b^	25.90 ± 0.22 ^b^	23.94 ± 0.35 ^a^	24.18 ± 0.12 ^a^
Appearance	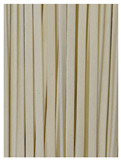	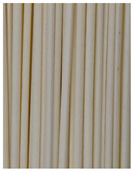	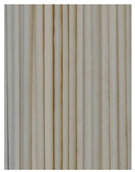	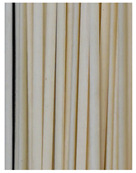	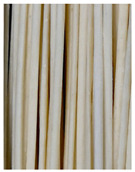
	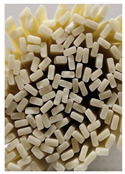	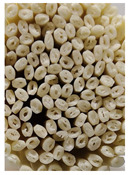	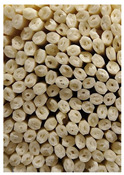	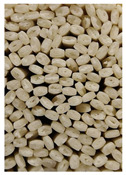	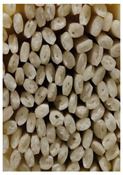
Size	1.1 mm × 2.3 mm	1.6 mm × 2.1 mm	1.5 mm × 2.1 mm	1.6 mm × 2.1 mm	1.7 mm × 2.1 mm

CT, Cooking time; CL, Cooking loss; Control, unfermented noodles; HN1, hollow noodles 1; HN2, hollow noodles 2; LPHN, *Lactobacillus plantarum*-fermented hollow noodles; KJHN, Koji-fermented hollow noodles. All data represent the mean of triplicates. Means in the same row with different letters indicate a significant difference at *p* < 0.05.

**Table 2:** Thermal properties, crystallinity and short-range order degree of starch from dried noodles.

Sample	Thermal Properties				Relative Crystallinity %	Short-Range Order Degree
	T_0_ (°C)	T_p_ (°C)	T_c_ (°C)	ΔH (J/g)		FWHM
Control	54.92 ± 0.12 ^b^	63.67 ± 0.23 ^c^	71.75 ± 0.82 ^b^	5.17 ± 0.21 ^b^	20.59 ± 0.40 ^b^	19.48 ± 0.12 ^b^
HN1	54.92 ± 0.59 ^b^	63.94 ± 0.10 ^c^	73.00 ± 0.83 ^b^	5.16 ± 0.19 ^b^	20.36 ± 0.20 ^b^	19.36 ± 0.16 ^b^
HN2	54.92 ± 0.12 ^b^	63.83 ± 0.00 ^c^	73.50 ± 0.71 ^b^	5.25 ± 0.13 ^b^	20.60 ± 0.34 ^b^	19.25 ± 0.30 ^b^
LPHN	55.72 ± 0.25 ^a^	65.83 ± 0.54 ^a^	75.17 ± 0.73 ^a^	6.80 ± 0.24 ^a^	22.19 ± 0.38 ^a^	18.60 ± 0.14 ^a^
KJHN	55.42 ± 0.83 ^a^	65.20 ± 0.47 ^b^	76.50 ± 0.71 ^a^	6.86 ± 0.28 ^a^	21.86 ± 0.54 ^a^	18.52 ± 0.13 ^a^

T_0_, starting temperature; T_p_, peak temperature; T_c_, end temperature; ΔH, enthalpy; Control, unfermented noodles; HN1, hollow noodles 1; HN2, hollow noodles 2; LPHN, *Lactobacillus plantarum*-fermented hollow noodles; KJHN, Koji-fermented hollow noodles. FWHM: full width at half maximum. All data represent the mean of triplicates. Means in the same column with different letters indicate a significant difference at *p* < 0.05.

## Data Availability

The dataset of the current study is available from the corresponding authors on reasonable request.
